# Identification of AIM2 as a downstream target of JAK2V617F

**DOI:** 10.1186/s40164-016-0032-7

**Published:** 2016-01-28

**Authors:** Ei Leen Liew, Marito Araki, Yumi Hironaka, Seiichi Mori, Tuan Zea Tan, Soji Morishita, Yoko Edahiro, Akimichi Ohsaka, Norio Komatsu

**Affiliations:** 1 Department of Hematology, Juntendo University School of Medicine, 2-1-1 Hongo, Bunkyo-ku, Tokyo, 113-8421 Japan; 2 Fujii Memorial Research Institute, Otsuka Pharmaceutical Co., Ltd., Shiga, Japan; 3 Department of Transfusion Medicine and Stem Cell Regulation, Juntendo University School of Medicine, Tokyo, Japan; 4 Division of Cancer Genomics, Cancer Institute of Japanese Foundation for Cancer Research, Tokyo, Japan; 5 Cancer Science Institute of Singapore, National University of Singapore, Singapore, Republic of Singapore

**Keywords:** Myeloproliferative neoplasms, Polycythemia vera, Essential thrombocythemia, Primary myelofibrosis, JAK2V617F, AIM2, IL1B

## Abstract

**Background:**

The gain-of-function mutation JAK2V617F is frequently found in Philadelphia-chromosome-negative myeloproliferative neoplasm (MPN) patients. However, the tumorigenic properties of JAK2V617F have mostly been characterized in in vivo and in vitro murine models due to the lack of appropriate human cell lines.

**Methods:**

Using the multipotent hematologic cell line UT-7/GM, we established D9, a novel human cell line that expresses JAK2V617F upon tetracycline addition. We assessed cellular differentiation in UT-7/GM cells when JAK2V617F was induced, and we used microarrays to analyze changes in mRNA expression caused by JAK2V617F.

**Results:**

Using the human D9 cell line, we demonstrated that the induction of JAK2V617F leads to cytokine-independent cell growth with increased STAT activation and erythroid differentiation, mimicking the characteristics observed in polycythemia vera, making it a suitable in vitro model for studying this disorder. Interestingly, JAK2V617F-dependent erythroid cell differentiation was blocked when GM-CSF was added to the culture, suggesting that the GM-CSF pathway antagonizes JAK2V617F-induced erythroid cell differentiation. Our microarray analysis identified several genes involved in inflammasome activation, such as AIM2, IL1B, and CASP1, which were significantly up-regulated in JAK2V617F-induced cells.

**Conclusions:**

The observed inflammasome activation following JAK2V617F induction is consistent with a recent report demonstrating the involvement of IL1B in myelofibrosis development in a JAK2V617F model mouse. These results indicate that the D9 cell line should be useful for characterizing the signaling pathways downstream of JAK2V617F, allowing for the identification of effector molecules that contribute to the development of MPN.

**Electronic supplementary material:**

The online version of this article (doi:10.1186/s40164-016-0032-7) contains supplementary material, which is available to authorized users.

## Background

Philadelphia-chromosome-negative myeloproliferative neoplasms (MPNs) include a range of clonal hematological malignant diseases such as polycythemia vera (PV), essential thrombocythemia (ET), primary myelofibrosis (PMF), and chronic neutrophilic leukemia (CNL). Unlike CNL that is associated with CSF3R mutation [1–3], PV, ET, and PMF share a common acquired mutation, JAK2V617F, which is found in approximately 95 % of PV patients and 50 % of ET and PMF patients [4–7]. This mutation somatically acquired in myeloid progenitor or hematopoietic stem cells [8] plays a causal role in MPN development in vivo (reviewed in [9]) and is thus defined as a driver mutation.

As opposed to wild-type JAK2, which is only activated when recruited to cytokine-bound cytokine receptors, JAK2V617F is constitutively activated through binding to the cytokine receptor, even in the absence of cytokines. JAK2V617F activation stimulates several downstream signaling networks, including the STAT3, STAT5, ERK/MAP kinase, and PI3 kinase/Akt pathways, leading to cytokine-independent cell growth (reviewed in [10]). Although activation of these pathways can partially explain the phenotypes observed in MPN patient cells, such as hypersensitivity to erythropoietin (EPO), it remains unclear how a single mutation can cause three distinct clinical phenotypes in humans [11].

Although human cell lines with the JAK2V617F mutation have been established from patients who had leukemic transformation from ET [12] or erythroleukemia [13], the use of these cell lines to study JAK2V617F function is limited by the leukemic transformation and concomitant loss of cytokine responsiveness. Therefore, studies of this mutation have been largely carried out using murine cell lines, and no other human cell lines carrying JAK2V617F have since been established. In this study, we established a novel human cell line with inducible JAK2V617F expression to determine how this mutation affects the regulation of other genes/networks downstream of JAK2. We used UT-7/GM as the basis for the cell line, which has the unique ability to differentiate into erythrocytes or megakaryocytes, depending on the presence of EPO or thrombopoietin (TPO), respectively [14, 15]. These cells will be an invaluable tool with which to investigate the downstream effectors of JAK2V617F, which should allow us to elucidate the relationship between MPNs and the JAK2V617F mutation.

## Methods

### Plasmid construction

**Fig. 1:**
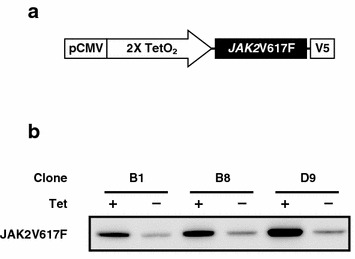
Creation of the Tet-inducible JAK2V617F-expressing cell line D9. **a** A schematic representation of the Tet-inducible construct containing the JAK2V617F gene tagged with a V5 epitope. **b** Confirmation of JAK2V617F induction. Equal amounts of protein prepared from clones treated with Tet or vehicle alone for 24 h were subjected to immunoblotting analysis using an anti V5 antibody to detect JAK2V617F

A 3.4 kb JAK2 cDNA with a G2490A substitution causing the V617F mutation was amplified by PCR and subcloned using TOPO-cloning into pcDNA3.1D (Invitrogen, Carlsbad, CA, USA). Using PCR and subsequent subcloning, the stop codon in the JAK2 cDNA was deleted, resulting in the addition of a V5-tag with a spacer sequence (derived from the vector) at the carboxy-terminal of JAK2. The V5-tagged JAK2V617F cDNA was excised using PmeI and SacI digestion, blunted, and then inserted into the PmeI site of pcDNA4/TO (Invitrogen) to generate the final Tet-inducible construct (Fig. 1a).

### Establishment of a Tet-inducible cell line

The parental cell line UT-7/GM/TetR, which constitutively expresses the Tet-repressor (TetR) [16], was cultured in Iscove’s Modified Dulbecco’s Media (IMDM) supplemented with 10 ng/ml GM-CSF, 10 % (v/v) heat-inactivated Tet-free fetal bovine serum (FBS) (Takara Bio Company, Otsu, Japan), and penicillin–streptomycin at 37 °C in a 5 % CO_2_ incubator.

**Fig. 2:**
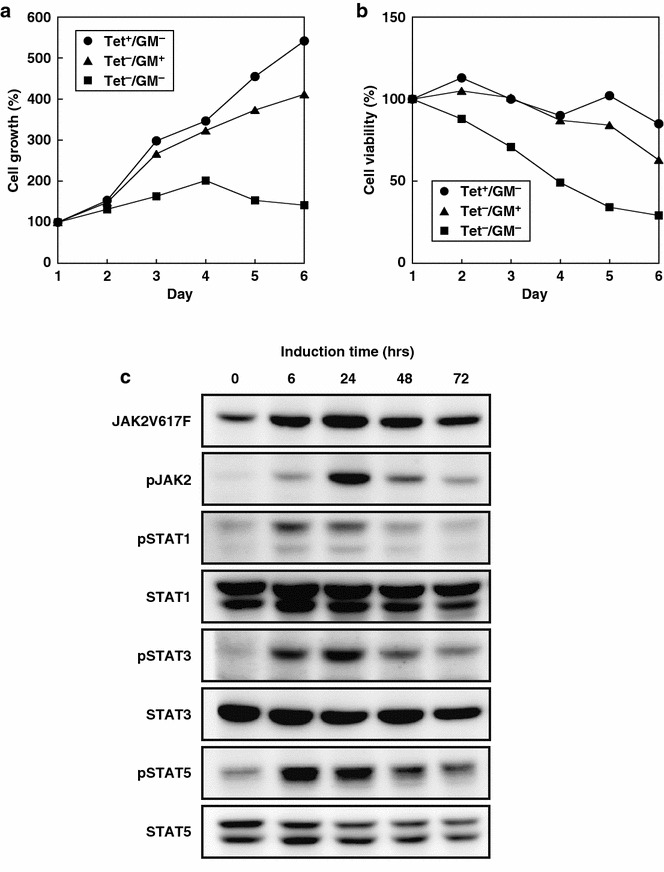
Cytokine-independent growth of D9 cells through JAK2 and STAT-activation. **a** A cell growth curve for D9 cells cultured in the presence of GM-CSF (*GM, triangle*) or Tet (*circle*) supplemented every other day, or in the absence of both GM-CSF and Tet (*square*). Cell numbers were counted daily for the indicated period, and growth relative to day 1 for each condition is shown. **b** At the same time, cell viability was also assessed using a dye exclusion assay, and representative samples are shown. **c** JAK2 and STAT activation upon JAK2V617F induction. Equal amounts of protein from cells treated with Tet for the indicated periods were analyzed by immunoblotting analysis with the indicated antibodies. Note that the level of JAK2V617F and phosphor-STATs were decreased in 48 and 72 h compared to 24 h due to degradation of Tet, which was only added in the media at time 0

The construct carrying the JAK2V617F gene was introduced into UT-7/GM/TetR cells using Amaxa^®^ Nucleofector (Lonza, Basel, Switzerland) based on the protocol for nucleofection of suspension cell lines. The transfected cells were cultured for 48 h in 100 mm petri dishes, and then distributed into 24-well plates containing fresh medium with 200 µg/ml zeocin (Invitrogen) to select cells. Resistant cells in each well were independently transferred into 6-well plates to screen for cells that induced JAK2V617F. The cells were cultured with or without Tet (1 µg/ml) (Sigma-Aldrich, St. Louis, MO, USA) for 24 h before harvesting to prepare whole cell lysates for immunoblotting analysis. Positive clones that showed Tet-inducible JAK2V617F expression were selected and used to create single-cell colonies in methylcellulose-based medium. Individual colonies were picked and cultured for another round of immunoblotting screening for Tet-dependent induction. Positive clones were then stored at −80 °C. Cell proliferation and viability (Fig. 2a, b) were determined using the trypan-blue dye exclusion assay with a TC10 Automated Cell Counter (Bio-Rad, Hercules, CA, USA).

### Immunoblotting analysis

To prepare cell extracts for immunoblotting analysis, cells were washed twice with PBS containing 1 mM orthovanadate and then lysed with lysis buffer CelLytic M (Sigma-Aldrich) supplemented with a protease and phosphatase inhibitor cocktail (Thermo Scientific, Waltham, MA, USA, Cat#78440) under vigorous shaking on ice for 30 min. Protein concentration was determined using a BCA Protein Assay Kit (Thermo Scientific). Equal amounts of protein were denatured, electrophoresed, and transferred to a PVDF membrane (Millipore, Billerica, MA, USA). For detection, a mouse monoclonal anti-V5 antibody (Life Technologies, Carlsbad, CA, USA) was used. Other antibodies used to detect related signals included phospho-JAK2 (Tyr1007/1008) (CST#3771), phospho-STAT1 (CST#9171), phospho-STAT3 (CST#9145), phospho-STAT5 (CST#9359), JAK2 (CST#3230), STAT1 (CST#9172), STAT3 (CST#9132), and STAT5 (CST#9363), which were purchased from Cell Signaling Technology (Danvers, MA, USA). The horseradish peroxidase-conjugated secondary antibodies used were a polyclonal rabbit anti-mouse IgG (DAKO, Santa Clara, CA, USA, #Z0259) for the mouse monoclonal anti-V5 antibody and a goat anti-rabbit IgG (Santa Cruz, Dallas, TX, USA, #sc-2004) for other primary antibodies. The chemiluminescence reaction was performed using the Pierce ECL Western Blotting Femto reagents (Thermo Scientific), and images were captured using LAS-3000 or LAS-4000 scanners (Fuji, Tokyo, Japan).

### *O*-dianisidine staining

D9 cells cultured for a week under various conditions were spun down, washed once with PBS, and then resuspended with serum-free RPMI. Next, a one-tenth volume of freshly prepared *O*-dianisidine staining solution (18.2 mg/mL *O*-Dianisidine/2.7 % hydrogen peroxide/2.7 % acetic acid) was added to each of the cell suspensions, and the cell mixtures were incubated at room temperature for 30 min. Cells were finally collected on glass slides via cytospin, and brown cells were counted by microscopy.

### Gene expression profiling and pathway analysis

RNA was isolated with TRIzol Reagent (Invitrogen) and purified using the Pure link RNA Mini Kit (Invitrogen) based on the manufacturer’s protocol. Microarray samples were prepared using 3′-IVT Expression Kit (Affymetrix, Santa Clara, CA, USA) and hybridized to a U133 microarray (Affymetrix), according to the manufacturer’s instructions. Following RMA normalization, significant differentially expressed genes were identified as the overlap between lists created using significance analysis of microarrays (SAM, q = 0) [17] and the receiver operating characteristic (ROC, pAUC = 1) [18]. For the pathway analysis, ssGSEA was applied to compute MSigDB v3.0 pathway enrichment scores for the samples [19, 20]. Subsequently, SAM/ROC was applied to the enrichment scores to identify significantly up-regulated pathways (q = 0, pAUC = 1). Up-regulated pathways were further evaluated based on information in the KEGG database (http://www.genome.jp/kegg/pathway.html).

Quantitative RT-PCR (qRT-PCR) was performed as described previously [21]. The qRT-PCR conditions used were 95 °C for 1 min, 45 cycles of 95 °C for 15 s, and 60 °C for 30 s. The following primers were used for RT-PCR: JAK2 (forward: 5′-TCTGGGGAGTATGTTGCAGAA-3′, reverse: 5′-AGACATGGTTGGGTGGATACC-3′); and AIM2 (forward: 5′-CAGACCCGGTTTGCTGAT-3′, reverse: 5′-TTACTCTCCATCTGACAACTTTGG-3′).

## Results

### Establishment of a JAK2V617F inducible cell line

To study the biological roles of the JAK2V617F oncogene product, we established a cell line in which JAK2V617F expression can be controlled by the addition of Tet. We employed a multipotent hematologic cell line, UT-7/GM [14, 15], so that we could assess the effects of JAK2V617F expression on both cell proliferation and differentiation. Using transfection and subsequent random insertion into the genome, we introduced a V5-tagged version of JAK2V617F cDNA under control of the Tet-ON promoter (Fig. 1a) into UT-7/GM/Tet-R cells that constitutively expressed Tet-R [16]. Cells bearing the expression construct were selected by culturing in zeocine-containing media, cloned from individual colonies in methylcellulose media, and then further screened for Tet-dependent induction of V5-tagged JAK2V617F using immunoblotting analysis. We isolated three independent clones: B1, B8, and D9. As D9 showed the strongest JAK2V617F induction following Tet addition (Fig. 1b), we employed this clone in subsequent analyses.

### Cytokine-independent cell growth of the D9 cell line with STAT activation

As JAK2V617F can activate the JAK-STAT pathway in the absence of cytokines in the murine pro-B cell line, resulting in cytokine-independent cell growth [22, 23], we asked whether the same phenomenon occurred in D9 cells. We cultured D9 cells in the absence of GM-CSF, an essential cytokine for the parental UT-7/GM cell line, with or without Tet addition for JAK2V617F induction. As shown in Fig. 2a, in the presence of Tet, D9 cells grew normally despite the absence of GM-CSF, whereas cell proliferation was strongly suppressed in the absence of Tet or GM-CSF. The viability of cells remained high for 5 days in Tet- or GM-CSF-supplemented culture, whereas removal of both factors resulted in significant reductions in cell viability (Fig. 2b). These findings demonstrate that the D9 cell line promotes cytokine-independent cell proliferation due to the Tet-induced expression of JAK2V617F.

To investigate whether JAK2V617F induction activates the JAK-STAT pathway in D9 cells, we examined the phosphorylation status of three important downstream molecules of JAK signaling: STAT1, STAT3, and STAT5. D9 cell extracts were prepared at different time points after Tet addition and subjected to immunoblotting analysis. In response to JAK2V617F induction (which reached maximum levels 24 h after Tet addition), we observed increased phosphorylation for STAT1, 3, and 5, as well as JAK2 in D9 (Fig. 2c) but not in parental UT-7/GM/TetR cells (Additional file 1: Figure S1). This evidence suggests that D9 cells acquire cytokine-independent cell growth at least in part, if not all, through activation of the JAK-STAT signaling pathway.

### JAK2V617F induction promotes erythroid cell differentiation in D9 cells

**Fig. 3:**
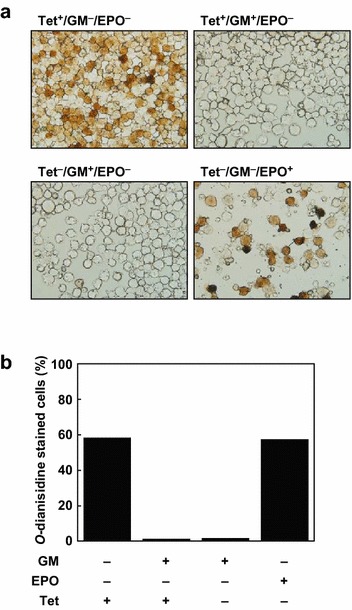
Induction of erythroid differentiation by JAK2V617F expression. **a** Images of *O*-dianisidine-stained D9 cells cultured under the indicated conditions for 7 days. **b** *Bar graph* representing the percentage of cells stained by *O*-dianisidine in each of the indicated conditions

Next, we examined whether JAK2V617F induction affects cell lineage determination in D9 cells, as the parental cell line is multipotent [15]. D9 cells were cultured under four different conditions: (1) Tet, (2) Tet and GM-CSF, (3) GM-CSF, and (4) EPO. After a week of culturing, cells were subjected to *O*-dianisidine staining (Methods) (Fig. 3a); stained and unstained cells were counted, and the results were plotted on a bar graph (Fig. 3b). Cells expressing JAK2V617F under the control of Tet differentiated to erythroid cells at levels similar to what was seen in the EPO-treated cells (Fig. 3). By contrast, in the presence of GM-CSF, cells remained undifferentiated, even in the presence of Tet. These data suggest that JAK2V617F induction itself promotes erythroid differentiation and that JAK2V617F-dependent differentiation is blocked by GM-CSF. The EPO-independent cell growth and the induction of erythroid differentiation by JAK2V617F in the D9 cell line suggests that this cell line has the same characteristics of PV following induction, making it suitable for use as an in vitro model to study PV.

### Identification of AIM2 as a downstream target of JAK2V617F

**Fig. 4:**
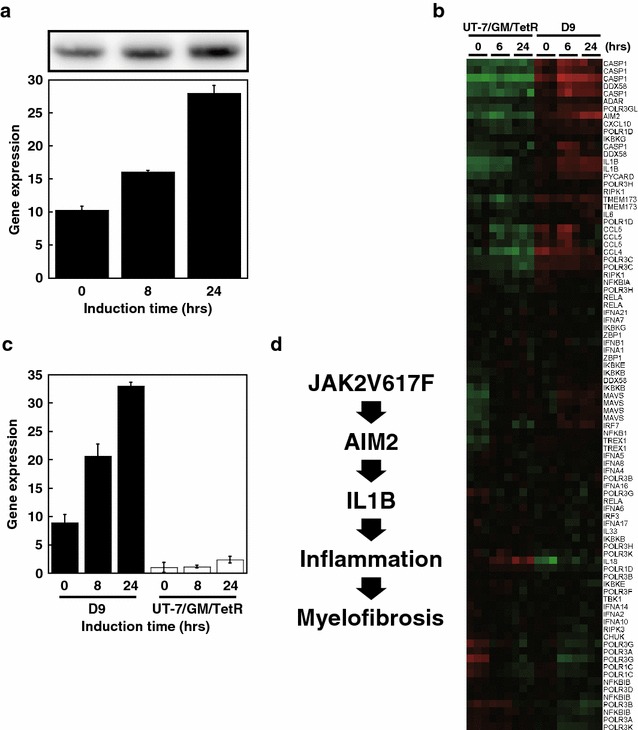
Identification of AIM2 as a downstream target of JAK2V617F. **a** Presumptive JAK2V617F induction was verified by qRT-PCR analysis for JAK2 at the indicated time points after Tet induction, as shown in the lower panel. V5-tagged JAK2V617F induction was observed by immunoblotting analysis (*upper panel*). Note that qRT-PCR did not discriminate between endogenous JAK2 and Tet-induced V5-tagged JAK2V617F mRNA, thus the rate of JA2V617F mRNA induction may be underrepresented. **b** A *heat map* showing the expression of genes in the cytosolic DNA sensing pathway in D9 and UT-7/GM/TetR (control) cells at the indicated time points. RNA samples from three independent experiments were analyzed. *Green* indicates lower gene expression and *red* indicates higher gene expression. **c** qRT-PCR analysis of AIM2 expression using the cDNA analyzed in (**b**). **d** A model explaining the potential roles of how AIM2 and IL1B might act downstream of JAK2V617F to contribute to myelofibrosis

To investigate the transcriptional cascade downstream of JAK2V617F, we performed a microarray-based mRNA expression analysis followed by single-sample gene set enrichment analysis (ssGSEA). Total RNA and cell lysates were prepared from D9 or UT-7/GM/TetR (control) cells that were subjected to a 3-h starvation period without GM-CSF, and then cultured in the presence of Tet for 0 (control), 6, or 24 h. The induction of JAK2V617F expression was confirmed by qRT-PCR and immunoblotting analysis (Fig. 4a). Then, RNA samples obtained from three independent experiments were subjected to microarray analysis. The pathway enrichment scores computed by ssGSEA based on SAM/ROC were compared between the D9 datasets at 6 and 24 h of Tet induction (JAK2V617F-induced D9) and the datasets for UT-7/GM/TetR (all time points) and D9 with no Tet induction (control). Among the 21 KEGG pathways that were classified as up-regulated in D9 cells (data not shown), we identified a cytosolic DNA sensing pathway that is involved in inflammasome activation. In particular, the genes associated with inflammasome activation, such as AIM2, CASP1, and IL1B, were strongly induced by JAK2V617F induction (Fig. 4b).

In clinical, PMF patients present increased level of pro-inflammatory cytokines such as IL1B [24]. Because AIM2 is reported to play an important role in IL-1B regulation [25, 26] and significantly induced in our assay (Fig. 4b), we further confirmed the up-regulation of AIM2 by JAK2V617F using qRT-PCR. As shown in Fig. 4c, we observed a nearly fourfold increase in AIM2 gene expression at 24 h of Tet induction relative to the 0 h (control). Therefore, we concluded that AIM2 is a downstream target of JAK2V617F in D9 cells.

## Discussion

In the present study, we describe the creation of a cell line, D9, which contains a tetracycline-inducible form of the JAK2V617F cDNA and was based on a subline of the acute megakaryoblastic leukemia UT-7 cell line. The induction of JAK2V617F in D9 cells promotes phosphorylation of downstream effector proteins such as STAT1, STAT3, and STAT5, leading to GM-CSF-independent growth and the induction of erythroid differentiation. Using a microarray analysis and ssGSEA, we identified a group of genes in the inflammasome pathway that were significantly up-regulated in JAK2V617F-induced cells. The robust induction of AIM2, a key component of the pathway, was confirmed by qRT-PCR, suggesting that AIM2 may be a downstream effector of JAK2V617F and may play a role in the development of myelofibrosis (discussed below).

Consistent with previous reports in a murine cell line [22, 23], the expression of JAK2V617F promotes STAT activation and subsequent cytokine-independent growth in human leukemia UT-7/GM cells (Fig. 2). Although some leaky expression of JAK2V617F (in the absence of Tet) was detected by our immunoblotting analyses (Figs. 1b, 2c, 4a), that level of JAK2V617F was not sufficient to generate GM-CSF-independent D9 cells, presumably due to the counteracting effects of the two copies of wild-type JAK2 [27] against the relatively weak JAK2V617F [28].

The UT-7/GM cell line is capable of differentiating into megakaryocytic and erythroid lineage cells in response to TPO and EPO, respectively. In D9 cells, however, the induction of JAK2V617F in the absence of GM-CSF results in differentiation towards the erythroid lineage (Fig. 3). This phenomenon is likely due to the high levels of JAK2V617F, as increased JAK2V617F allele frequencies and expression levels have been linked to cellular differentiation towards erythroid lineages in both MPN patients and murine models [9, 29, 30]. However, we note that UT-7/GM cells respond quicker to EPO compared with TPO, as judged by EPO receptor (EPOR) and TPO receptor (c-MPL) expression [15], indicating that UT-7/GM cells are predisposed to differentiate towards an erythroid cell lineage.

We observed that GM-CSF blocked the JAK2V617F-induced erythroid cell differentiation in D9 cells (Fig. 3), consistent with our previous finding that GM-CSF counteracts EPO signaling pathway in UT-7/GM cells, the parental cell line of D9 [15]. In addition, it was reported that JAK2V617F requires cytokine receptor binding for the activation of downstream pathway [31]. These observations suggest that although JAK2V617F is capable of transforming cells in a certain context, its function is restricted by counteracting signaling cascades related to cell linage. Thus, this mutation is predominantly found in PV, ET, and PMF patients who exhibit aberrant erythrocytosis and/or megakaryocytosis, but not commonly in other myeloproliferative neoplasms such as CNL and chronic myeloid leukemia. Further understanding context-dependent oncogenic functions of JAK kinases are beneficial for the development of novel therapeutic strategies to overcome issues in the treatment of MPN [32] and other hematopoietic malignancies [33]. Nevertheless, the JAK2V617F-induced cytokine-independent cell proliferation and erythroid differentiation exhibited by D9 cells in this study demonstrates the two key features of PV, making it a suitable in vitro model for further studies of the causal role of JAK2V617F.

Using mRNA microarray analysis and subsequent ssGSEA, we identified a cytosolic DNA-sensing pathway that includes genes involved in formation of the inflammasome (Fig. 4). Basal AIM2 expression in non-induced D9 cells was significantly higher than in UT-7/GM/TetR control cells (Fig. 4c), suggesting that leaky JAK2V617F expression is sufficient to induce AIM2 expression, which was increased further in Tet-induced D9 cells (Fig. 4c). As AIM2 plays a crucial role, along with CASP1, in converting pro-IL1B to its active form [25, 26], the induction of the AIM2, CASP1, and IL1B mRNAs in JAK2V617F-induced cells suggests that IL1B activation is linked to MPN development. More recently, IL1B secreted from JAK2V617F-positive hematopoietic stem cells has been shown to induce inflammation and promote the development of myelofibrosis in the bone marrow in animal models [34]. Our microarray analysis also revealed increased levels of DDX58 mRNA, another component of the cytosolic DNA sensing pathway (Fig. 4b). DDX58 was previously identified as one of the MPN-associated genes that exhibits genomic amplification at chromosome 9 in MPN patients, compared with normal healthy individuals [35]. This suggests that increases in DDX58 gene dosage could contribute to MPN pathogenesis. Although we successfully identified genes of which expression is induced by JAK2V617F in D9 cells, we note that the continuous leaky expression of JAK2V617F may alter the cellular property and its response to JAK2V617F. Further analysis using more defined assay system is required for gaining clearer and definitive view on genes regulated by JAK2V617F. Finally, we propose a model in which high levels of JAK2V617F induce the expression of inflammasome-related genes, triggering myelofibrosis development in the bone marrow, which has been associated with increased JAK2V617F allele frequencies in patients [4, 6, 36, 37] and high levels of mutant expression in animal models [9] (Fig. 4d).

## Conclusions

The induction of JAK2V617F expression in D9 cells promoted cell proliferation in the absence of a growth cytokine (GM-CSF) and led to erythroid differentiation, making this human cell line-based model useful for studying the causal effects of the JAK2V617F mutation on MPN development. Using D9 cells, we identified several genes involved in the inflammasome pathway, such as AIM2, IL1B, and CASP1, as downstream targets of JAK2V617F, which could explain the molecular mechanism behind myelofibrosis development in patients harboring the JAK2V617F mutation. Further studies to elucidate the activities of these downstream signals and pathways should shed light on MPN development, which could lead to the creation of drugs targeting the effectors of the JAK2V617F mutation.

## Additional file

**Additional file 1: Figure S1.** No inductionon of pJAK2 and pSTAT3 in the parental UT-7/GM/TetR cells by Tet.

## Abbreviations

MPN: myeloproliferative neoplasms
Tet: tetracycline
PV: polycythemia vera
ET: essential thrombocythemia
PMF: primary myelofibrosis
EPO: erythropoietin
TPO: thrombopoietin
TetR: Tet-repressor
IMDM: Iscove’s Modified Dulbecco’s Media
FBS: fetal bovine serum
SAM: significance analysis of microarrays
ROC: receiver operating characteristic
ssGSEA: single-sample gene set enrichment analysis
qRT-PCR: quantitative RT-PCR
