# Supplementary material for: Identification of AIM2 as a downstream target of JAK2V617F
Source: Exp Hematol Oncol. 2016 Jan 28;5:2. doi: 10.1186/s40164-016-0032-7 (PMC4730608; doi:10.1186/s40164-016-0032-7)
Supplement: Supplementary file 1 — Additional file 1: Figure S1. No inductionon of pJAK2 and pSTAT3 in the parental UT-7/GM/TetR cells by Tet. [file 40164_2016_32_MOESM1_ESM.pdf]

Liew et al.

Supplemental Data

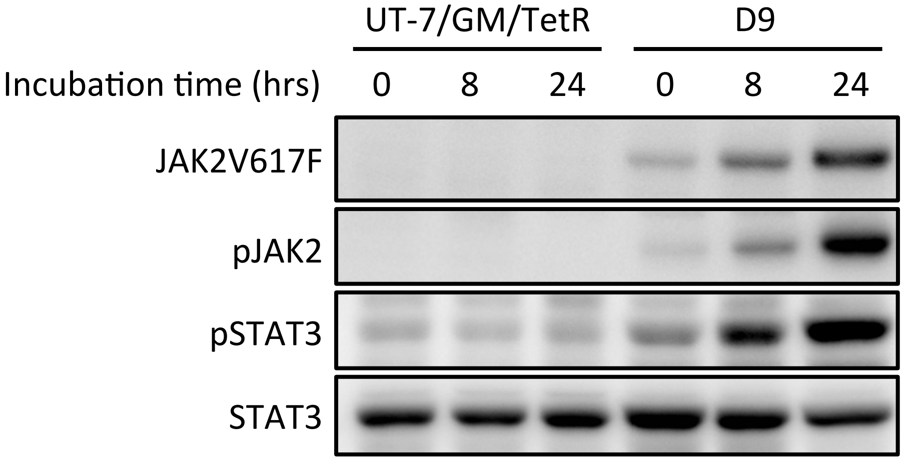

Supplemental Figure 1. No induction of pJAK2 and pSTAT3 in the parental UT-7/GM/TetR cells by Tet.
JAK2 and downstream molecule activation is induced by Tet in JAK2V617F-introduced D9 but not in parental UT-7/GM/TetR cells. Cells were first incubated in the absence of GM-CSF, and then Tet was added to the culture media at time 0. Cell extracts were prepared after incubation for the indicated period. Immunoblot analysis was performed as described in the main text.
